# Vegetation and Landscape Shift After Beaver Settlement in a Mountainous Area

**DOI:** 10.3390/biology14111603

**Published:** 2025-11-16

**Authors:** Rita Rakowska, Alina Stachurska-Swakoń

**Affiliations:** Department of Plant Ecology, Institute of Botany, Faculty of Biology, Jagiellonian University in Kraków, ul. Gronostajowa 3, 30-387 Kraków, Poland; rita.rakowska@alumni.uj.edu.pl

**Keywords:** *Castor fiber*, engineering activity, landscape ecology, plant communities, species reintroduction, vegetation change, water retention, Carpathians

## Abstract

Beavers are classified as ecosystem engineers because their activities can significantly alter environmental conditions. Vegetation and landscape changes, based on a series of vegetation maps and satellite images between 1994 and 2022, were studied in a mountain valley of a protected area in the Polish part of the Eastern Carpathians. Eighteen plant communities were identified before the beavers were released, with moist and wet communities covering 76.8% of the area. After 25 years of beaver presence, the vegetation changed: fresh communities decreased from 23% to 10%, and communities with grey alder disappeared. At the same time, the moist and wet communities expanded and new ones developed. Overall, the share of these communities increased to 89% of the area, with the dominant tall herb Filipendulo-Geranietum and *Menyanthes trifoliata* community. A distinctive feature was an increase in vegetation patchiness with a corresponding decrease in the evenness index. Landscape analysis revealed a 9.5% increase in the length of the streambed and fluctuations in the number of beaver ponds (11–25) and migration corridors (4–20). The number of corridors increased as the availability of grey alder decreased. The total area of the ponds exceeded 2200 m^2^, indicating their significant role in water retention and modifying microclimatic conditions.

## Simple Summary

Beavers significantly influence their environment, particularly in areas close to their habitats. This effect is especially notable in mountain streams, where their presence may not be immediately obvious. Their activities, such as dam construction, led to landscape changes in the small mountain valley in the Eastern Carpathians between 1994 and 2022. These included the creation of ponds and migration corridors, the number of which can vary from year to year. An indirect impact of beavers was the alteration in the length of the streambed, which increased by 9.5% due to the formation of meanders. The ponds created by beavers can exceed 2200 m^2^, contributing to local humidity levels. As the water accumulates behind the dams, soil moisture increases, resulting in changes in vegetation around the watercourse. Our findings, based on a unique series of vegetation maps, point to a decrease in fresh vegetation, the extent of moist and wet plant communities, a modification of their distribution within the valley, and an increase in vegetation patchiness. Over time, a dynamic vegetation mosaic can be observed that supports biodiversity.

## 1. Introduction

Living organisms that can change the environment, meeting their ecological needs and creating habitats for other organisms, are called ecosystem engineers. They can modify both biotic and abiotic components of their surroundings, leading to immediate or long-term effects [1,2,3].

Beavers are considered ecosystem engineers and are keystone species in wetland areas that support healthy functioning ecosystems by managing water resources through their building activity and feeding behaviour [4,5,6]. These positive outcomes of their activity encourage the intentional introduction of animals to help change the degraded environment to restore the proper ecological functions of ecosystems. Their activity can lead to hydrological buffering, increased habitat diversity, and the restoration of biodiversity [7,8].

Currently, the population of Eurasian beaver (*Castor fiber* L.) in the northern hemisphere is estimated to be approximately one and a half million individuals [9], although at the beginning of the 20th century, it was estimated to be only 1200 individuals across Eurasia as a result of over-hunting and habitat loss [9]. This rapid population growth was caused by efforts to reintroduce the beaver through reintroduction programmes in European countries, and then as a consequence of natural dispersal [9,10,11] and unofficial releases [12,13,14]. Such a large overall population brings about many effects on the environment and the human economy, assessed both positively (restoration of naturalness, increased biodiversity, and water retention) and negatively, understood as costs resulting from losses in agricultural economy, forestry, or urbanised areas [2,8,15,16,17,18,19]. In light of this information, it seems crucial to analyse beavers’ potential impact on the environment, the possibility of their coexistence with humans, and to have a good understanding of the ecological consequences of releasing such animals before deciding to introduce them to new locations [8,19,20].

Numerous detailed and review studies indicate the effects of beaver engineering activities. These include, among others, the influence on hydrology, e.g., refs. [21,22], geomorphology, e.g., refs. [23,24], and different aspects of plant and animal diversity, e.g., refs. [25,26,27,28]. The engineering activities of the Eurasian beaver (*Castor fiber* L.) lead to increased water level and soil moisture, e.g., refs. [29,30,31]. This, in turn, enhances biodiversity on a landscape scale, e.g., refs. [23,25,32,33,34], and supports the development of new plant communities that were previously absent in that area, e.g., refs. [18,19,20]. Despite growing knowledge of beavers’ impact on the environment, significant gaps remain, particularly with regard to their impact on flora and vegetation, as this type of research requires tedious and long-term investigations [19]. A review of beaver influence on vegetation [19] pointed out that many studies indicate a decrease in woody plant resources in areas influenced by beavers, e.g., refs. [15,16], and an increase in the coverage of moisture-loving herbaceous plants [25,26,35,36,37].

Our study aims to estimate the influence of beaver activity on vegetation and landscape in a specific mountain valley in the protected area, giving the opportunity to observe naturally occurring processes after introducing beavers and their long-term presence. We formulate the following hypotheses: (a) beaver activity leads to an expansion of the area occupied by non-forest wet and moist communities; (b) the total length of stream increases as a result of beaver construction; and (c) beaver presence increases water retention (measured by the surface area of ponds).

## 2. Materials and Methods

### 2.1. Study Area

**Figure 1:**
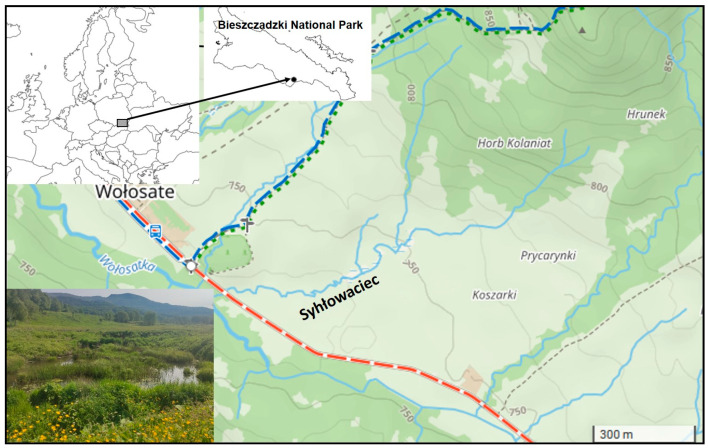
Location of the study area: Syhłowaciec stream valley in the Bieszczadzki National Park, Eastern Carpathians, Poland. (source: mapa-turystyczna.pl; changed).

The field study was conducted in the valley of the Syhłowaciec stream within the Bieszczadzki National Park in the Eastern Carpathians, Poland (Figure 1). The reintroduction programme of beaver settlement in the Bieszczady Mountains began in 1992, and the main objective of reintroduction was to restore the species to its historical range, improve local hydrological conditions by increasing water retention, restore habitats, and increase biodiversity in the upper San River valley, which had been degraded as a result of inappropriate agricultural practices carried out before the national park was established [37,38,39]. Between 1993 and 2003, 57 beaver families, a total number of 191 individuals, were reintroduced into the park area, preceded by a detailed selection of suitable locations [26]. The current population is estimated to be around 160 individuals [40,41]. This well-documented reintroduction followed extensive research [26] and was supported by historical data, as well as a long-term presence of beavers in the protected area. These factors have enabled the observation of changes in the beaver population [40,41], in the landscape [25,42,43], in the presence and appearance of plant species [25,26,41,44,45], and in animal species [25,27,46,47].

Beavers were introduced to the Syhłowaciec valley in 1996. Syhłowaciec is a small right tributary of the Wołosatka River, which is part of the San River catchment. The sources of the Syhłowaciec stream are located 800 metres above sea level on the southwestern slope of Tarnica Mountain, the highest peak in the Polish Bieszczady Mountains. The total length of the stream was primarily, i.e., before beavers were released, 1.44 km, and the catchment area was 0.4 km^2^ [28]. The Syhłowaciec stream flows into the Wołosatka at an altitude of 730 m. Beavers were released in the lower part of the valley and, until 2023, were observed along 58% of the stream’s length in the middle and lower part of the valley. The upper section of the stream has a distinctive mountainous character, featuring a narrow riverbed and relatively steep slopes, and runs through a forested area. To date, no beaver activity has been recorded in this upper part. Taking into account the terrain relief, previous observations of beaver activity, and information from the literature [48], the fieldwork was carried out in the middle and lower parts of the valley, focusing on an area situated 30–50 m from the centre of the Syhłowaciec stream, covering a total area of 6.7 hectares. Before the establishment of the national park, this part of the valley was used for agricultural purposes [46]. The valley lies in a moderately warm climate zone with a +6–+8 °C average annual temperature and about 1100 mm total annual precipitation [47]. Climate parameters changed between 1967 and 2016: total annual precipitation increased from 950 mm in 1967 to 1109 mm in 2016, and the average annual temperature increased by 2.23 °C from 7.08 °C in 1967 to 9.31 °C in 2016 [49].

### 2.2. Data Collection

#### 2.2.1. Vegetation

Detailed vegetation studies were performed in the middle and lower parts of the Syhłowaciec stream valley from 2018 to 2021 [50]. Ninety-two phytosociological relevés were established using the Braun-Blanquet method [51], each of 5 m × 5 m. The relevés represented all non-forest plant communities in the valley. During fieldwork, vegetation mapping was conducted using the following two methods: a printed orthophotomap from 2017 on a scale of 1:3000 and a Garmin GPSMAP 64 in the WGS84 system.

To analyse the influence of beaver activity on vegetation, all vegetation maps available in the area were examined. In total, vegetation maps from the following three periods were used: 1996, before beavers were released (the archive of BdNP), 2009–2010 (the archive of BdNP; ref. [52]), and the vegetation map made by the authors in 2018–2021. A detailed comparison of species changes during 1996–2009–2021 was not possible due to the limited number of phytosociological relevés.

#### 2.2.2. Landscape

To assess the landscape changes caused by beavers, we used available orthophotomaps of the Syhłowaciec stream valley that were at an appropriate resolution, enabling detailed analyses. In total, we analysed maps from the years 1994, 2009, 2015, 2017, 2019 (https://www.geoportal.gov.pl), and 2021 (archive BdNP). We accessed drone images from 2016 (archive BdNP). Additionally, we supplemented our material with aerial images from Google Maps, if they had sufficiently good resolution (Google Maps).

### 2.3. Data Analysis

#### 2.3.1. Vegetation

To identify the types of communities and the homogeneity of the relevés (plots) in the studied area, a classification method of the unweighted pair group (UPGMA) was used based on cover-abundance values with Jaccard and Ružicka coefficients (MVSP 3.22 [53]). The main clusters were determined following the syntaxonomical classifications provided by Matuszkiewicz [54], Kącki et al. [55], and Denisiuk and Korzeniak [56].

Non-metric multidimensional scaling with the Bray–Curtis coefficient was used to explore the similarity of the vegetation plots and their affinities (Past 5.2 [57]). Ecological Indicator Values (EIVs) for Poland, as outlined by Zarzycki et al. [58], were used to assess habitat conditions. The used EIVs are equivalent to Ellenberg EIVs; however, they should better reflect the ecological requirements of species occurring in south-eastern Poland [58]. The weighted means of the EIVs were calculated for each plot to estimate the environmental condition of the plots. We used the following climate parameters: light (L) and temperature (T), and the following soil parameters: soil moisture (W), soil trophy (Tr), and soil acidity (R). The discriminant analysis for EIVs was calculated to reveal the main factors that differentiate the types of plant communities (Statistica 13.3 software).

To evaluate alpha diversity of communities, several indices were calculated using MVSP 3.22 software, as follows: the Shannon–Wiener index (H), the evenness index (J), and the richness index (S). The significance of the diversity indices and EIVs was tested using the Kruskal–Wallis non-parametric test (Statistica 13.3 software).

To examine changes in beta diversity, vegetation maps from three periods were compared. The following parameters were analysed: the specific plant communities (including numbers and types), the occupied area (in ha and percentage of cover of the studied area), and the main types of plant communities according to soil moisture (habitat group), including fresh, moist, and wet. Before this analysis, the original cartographic materials were prepared appropriately to make analyses possible (e.g., facilitate comparison). Changes in occupied areas by community types and habitat groups were tested using the Wilcoxon signed-rank test.

#### 2.3.2. Landscape Changes

To analyse the changes in the landscape scale on the basis of the obtained maps and aerial images, we used ArcMap 10.3 software to analyse the data at scales of 1:1000 or 1:2500, depending on the detail needed. The analysis involved several functions, including rectification (drone images and Google maps), polygon and polyline creation, and shapefile measurement. In our study, we analysed the length of the stream, the number of beaver ponds, and the area covered by these ponds. For the length of the stream, the number of beaver ponds, and migration corridors, we used data from our previous research [59]. A beaver pond was defined as a visible water surface area that could be distinguished on an orthophotomap at a scale of 1:2500 and that exceeded the width of the streambed. Since the 2021 orthophotomap did not cover the upper part of the valley, we used mapping from previous years for that part of the study area. Changes in the pond surface between years were tested using the Wilcoxon signed-rank test. A migration corridor, i.e., a path built by beavers to enable them to move beyond the main stream [4], was defined as a visible line (with water) in the surrounding vegetation connected to beaver ponds.

We also used several landscape-scale metrics [60,61] based on vegetation maps and the ArcMap analyses results. For this purpose, we defined (I) habitat group as fresh, moist, and wet; (II) unit as the total area of a specific community; and (III) patch as an individual spot of community on the vegetation map surrounded by other community types. We used Patch Richness (PR): number of patches; Largest Patch Index (LPI): area dominance of the single largest patch (0–100%); Shannon–Wiener index (H): measure of landscape diversity using type and area of specific plant community; and Shannon’s evenness index (E): measure of dominance in landscape using type and area of specific plant community (0–1) (MVSP, [53]).

## 3. Results

### 3.1. Changes in Vegetation Under the Influence of Beaver Presence

**Figure 2:**
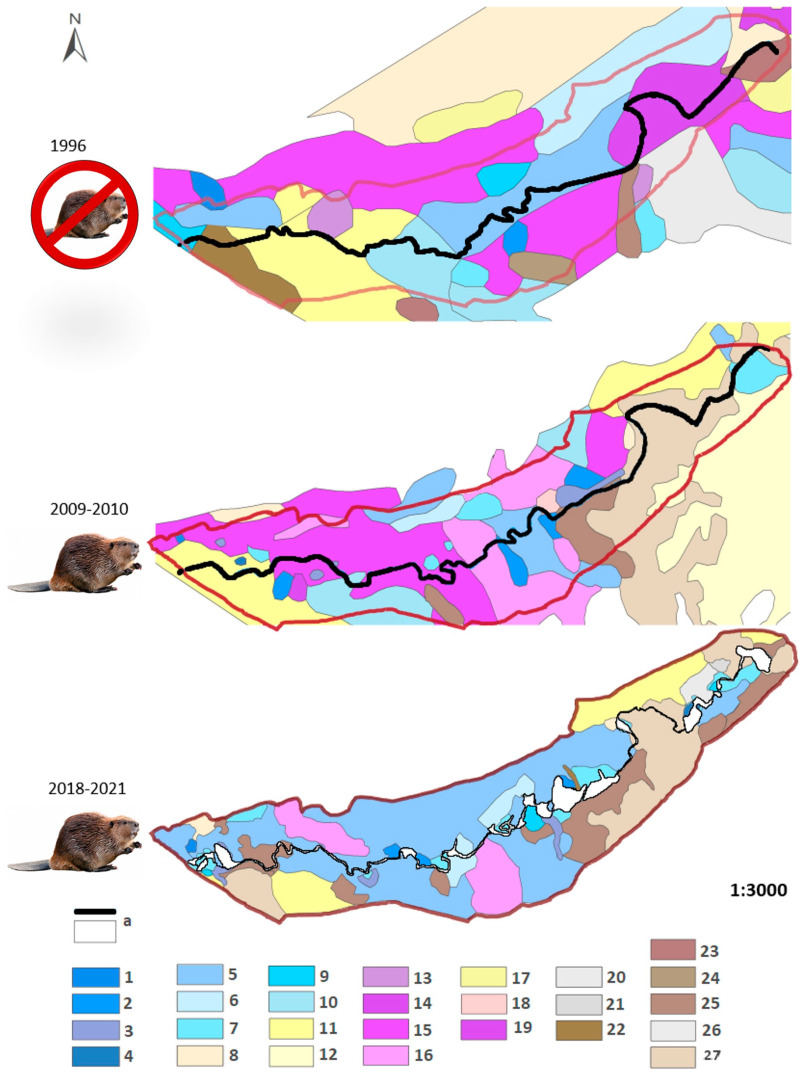
Vegetation maps of the Syhłowaciec stream valley, Bieszczadzki NP, Eastern Carpathians, 1996—before beaver release (source of map: BdNP archive); 2009–2010—14 years with beaver activity [51], 2018–2021—25 years with beaver activity. a—Syhłowaciec stream and beaver ponds; 1—Caricetum paniculatae, 2—Caricetum rostratae, 3—Equisetetum fluviatilis, 4—Sparganio-Glycerietum, 5—Filipendulo-Geranietum, 6—Cirsietum rivularis, 7—Scirpetum sylvatici, 8—community with *Deschampsia caespitosa*, 9—community with *Mentha longifolia*, 10—Epilobio-Juncetum effusi, 11—Campanulo serratae-Agrostietum capillaris alopecuretosum pratensis, 12—Campanulo serratae-Agrostietum typicum, 13—Caricetum diandrae, 14—Carici canescentis-Agrostietum caninae, 15—Valeriano-Caricetum flavae, 16—community with *Menyanthes trifoliata*, 17—Poo-Deschampsietum caespitosae, 18—community with *Vaccinium myrtillus*, 19—Caltho laetae-Alnetum, 20—Phalarido-Petasitetum hybridi, 21—Anthriscetum sylvestris, 22—community with *Urtica dioica*, 23—community with *Agropyron repens*, 24—community with *Calamagrostis canescens*, 25—community with *Carex brizoides*, 26—community with *Alnus incana* on agricultural land, 27—thickets.

During 25 years of beaver activity in the valley, changes in vegetation occurred both qualitatively, regarding the types of plant communities, and quantitatively, regarding the number of communities in particular periods and the area occupied by individual plant communities (Figure 2, Table 1). Between 1996 and 2021, a total of 26 plant communities were identified in the Syhłowaciec valley. These communities represent the habitat gradient from fresh to wet habitat and belong to the following syntaxonomical classes: Artemisietea vulgaris, Molinio-Arrhenatheretea, Betulo-Adenostyletea (=Mulgedio-Aconitetea), Phragmitetea, Scheuchzerio-Caricetea nigrae, Querco-Fagetea, and communities outside classification (Table 1).

Before beavers were released into the Syhłowaciec valley, eighteen plant communities were recorded, mainly with a patchy distribution. The distinctive characteristic for that time was the occurrence of fresh meadow Campanulo serratae-Agrostietum capillaris alopecuretosum, eutrophic mountain fen Valeriano-Caricetum flavae, tall herb Filipendulo-Geranietum, and a wet community with alder Caltho-Alnetum incanae (Figure 2, Table 1). After fourteen years with beavers in the valley, seven new plant communities developed, mainly connected with moist or wet habitats, from the classes of Phragmitetea and Scheuchzerio-Caricetea nigrae (Figure 2, Table 1). At the same time, eight plant communities disappeared. To this group belong communities with grey alder that were replaced with willow thickets. Some of the fresh communities disappeared (such as Poo-Deschampsietum, a community with *Agropyron repens* and *Urtica dioica*) or decreased in the occupied area. However, the changes also concerned moist and wet communities, such as a decrease in the area of the moist meadow Cirsietum rivularis.

After the following eleven years, during 2018–2021, sixteen plant communities were observed in the valley (Figure 2, Table 2). The largest area was occupied by the tall herb Filipendulo-Geranietum, which occurred mainly on the floodplain terrace. Eutrophic mountain fen Valeriano-Caricetum flavae disappeared, and in its place, a wet community with bogbean *Menyanthes trifoliata* developed. Wet communities with sedges extended their range, and, at the same time, the area of fresh meadows decreased (Table 1).

Ten plant communities were present throughout the study period. These included the following sedge beds: Caricetum paniculatae, Caricetum rostratae, Scirpetum sylvatici, the community with *Carex brizoides*, tall-herb Filipendulo-Geranietum, moist meadow Cirsietum rivularis, the community with *Deschampsia caespitosa*, and fresh meadow Campanulo serratae-Agrostietum capillaris alopecuretosum pratensis. Despite their long-term presence, both the coverage and locations of these communities changed over time (Table 1, Figure 2). Over the last 25 years, the area covered by the sedge and rush communities of the Phragmitetea class increased from 0.06 to 0.58 hectares, likely related to rising groundwater level. During the studies conducted in 2018–2021, the stream valley was predominantly covered by Filipendulo-Geranietum, with its coverage increasing significantly from 1.11 to 2.71 hectares. Despite the high moisture level in the soil, an increase in the area of the *Carex brizoides* community was also observed, increasing from 0.09 to 0.76 hectares. On the contrary, a decrease in coverage was observed for *Deschampsia caespitosa* and the community with *Menyanthes trifoliata*.

When comparing the types of communities over the years, there are notable differences in both the number of plant communities and their total coverage in the valley (Table 2). The number of plant communities decreased from 18 in 1996 to 16 in 2021. Although the counts of ongoing, extinct, and newly established plant communities remained the same (as shown in Table 2, the frequency of types was not statistically significant), the specific communities changed between study periods. For example, the community with *Mentha longifolia* disappeared between 1996 and 2010. However, this community reappeared in 2021 in an active beaver dam. Epilobio-Juncetum effusi was observed in both 1996 and 2010 in the studied area, but its presence was not confirmed in 2021.

**Figure 3:**
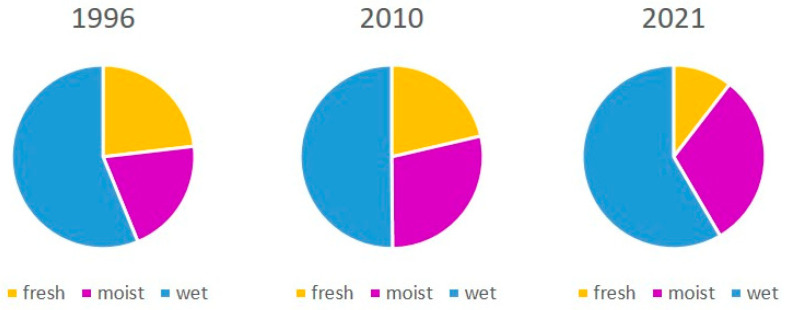
Percentage of cover of habitat group of plant communities in the Syhłowaciec stream, Bieszczadzki NP, Eastern Carpathians. 1996—before beaver release (source of map: BdNP archive); 2010—14 years with beaver activity [51], 2021—25 years with beaver activity.

Compared to vegetation before beavers were released in the valley, there was a statistically significant change in the cover of fresh and moist communities (Figure 3, Table 2). The area of fresh communities decreased from 23.15% in 1996 to 10.46% in 2021 (Wilcoxon test, *p* < 0.05). Similarly, the total area of moist communities increased from 20.65% in 1996 to 31.07% in 2021 (Wilcoxon test, *p* < 0.05). The total area occupied by wet communities also increased; however, the rise was not so spectacular, from 56.19% to 58.47%. The total area of moist and wet plant communities rose from 76.8% to 89.5%.

### 3.2. Diversity and Variability of Plant Communities

**Figure 4:**
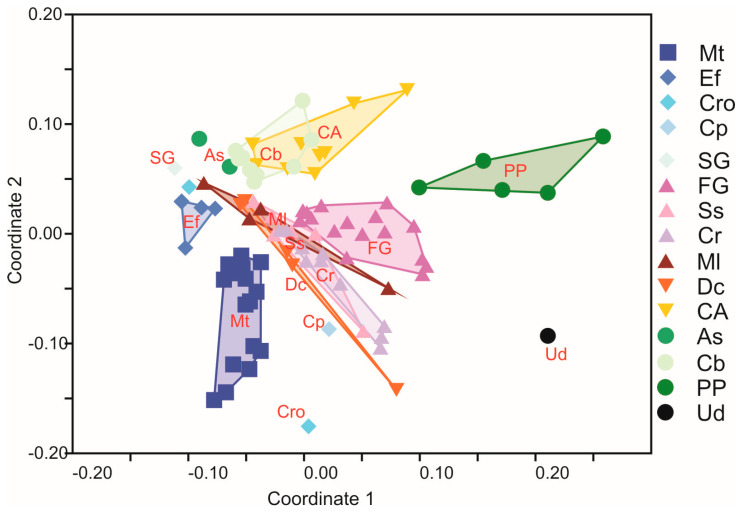
Non-metric multidimensional scaling of the vegetation plots in the Syhłowaciec valley in 2021, BdNP, Eastern Carpathians. Mt—community with *Menyanthes trifolium*, Ef—Equisetetum fluviatilis, Cro—Caricetum rostratae, Cp—Caricetum paniculatae, SG—Sparganio-Glycerietum, FG—Filipendulo-Geranietum, Ss—Scirpetum sylvatici, Cr—Cirsietum rivularis, Ml—community with *Mentha longifolia*, Dc—community with *Deschampsia caespitosa*, CA—Campanulo serratae-Agrostietum capillaris alopecuretosum pratensis, As—Anthriscetum sylvestris, Cb—community with *Carex brizoides*, PP—Phalarido-Petasitetum hybridi, Ud—community with *Urtica dioica*.

The non-metric multidimensional scaling of the vegetation plots in the Syhłowaciec valley revealed the similarity of the plots that represent the main types of plant communities and separated the communities that occurred in habitats with different moisture levels (Figure 4). According to discriminant analysis, soil fertility (Tr) is the most important environmental variable that influences the diversity of plant community types (Table 3).

The analysis of species diversity indices showed significant differences between all plant communities regarding species richness (S = 61.64, *p* < 0.01), Evenness index (J = 69.93, *p* < 0.01), Shannon–Wiener index (H = 62.67, *p* < 0.01), and Ecological Indicator Values (EIVs), as follows: light (L = 77.42, *p* < 0.01), temperature (T = 75.33, *p* < 0.01), soil moisture (W = 86.09, *p* < 0.01), soil fertility (trophy) (Tr = 77.24, *p* < 0.01), and soil acidity (R = 84.81, *p* < 0.01). In terms of species richness, the highest number of species was recorded in the fresh meadows of the Molinio-Arrhenatheretea class. For the evenness index, the associations Equisetetum fluviatilis, Caricetum rostratae, and Sparganio-Glycerietum fluitantis showed the highest levels of homogeneity, while Caricetum paniculatae, Filipendulo-Geranietum, and Cirsietum rivularis showed the least. The calculated mean EIVs revealed the greatest variation in soil moisture and soil acidity. The other mean values were generally high, indicating moderate light, moderately warm climatic conditions, and nutrient-rich soils. The highest mean values of soil moisture were recorded for the patches of the Phragmitetea class and the community with *Menyanthes trifoliata*. The associations of Filipendulo-Geranietum, Cirsietum rivularis, Scirpetum sylvatici, and the community with *Mentha longifolia* of the Molinio-Arrhenatheretea class were also linked to higher moisture levels. The mean soil acidity values were generally neutral, with one exception: the association of Caricetum rostratae.

### 3.3. Changes in the Landscape

**Figure 5:**
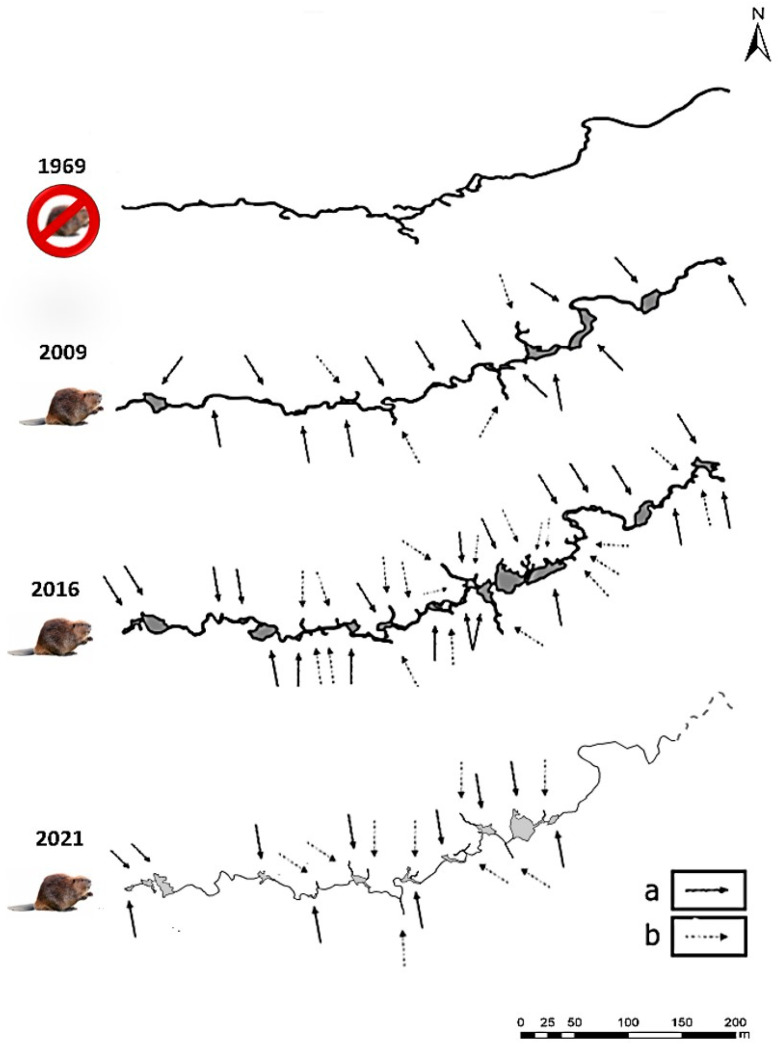
Changes in the streambed of the Syhłowaciec stream, 1969 (=1994)—stream without beaver presence; 2009, 2016, and 2021—stream with beaver presence. 2021—incomplete map for the analysis, upper part of the stream reproduced from previous years. a—an arrow indicates a beaver pond, b—an arrow indicates a migration corridor.

The analysis of orthophotomaps revealed that the attributes studied related to beaver activity, i.e., ponds (the number and surface), migration corridors, and the length of the stream, changed over time (Table 4 and Table 5, Figure 5). Over the 26 years since the introduction of beavers (1996–2022), the stream length, in measured parts, increased from 837 m in 1994 (which was the same as in 1969, before the beaver release) to 918 m in 2022. This represents an elongation of the stream course by 81 m, which is a 9.6% increase from the original length. A comparison of the stream’s appearance indicates that some sections altered their original course, contributing to this elongation (Figure 5). The analysis of the shapefiles shows that the meandering sections played a crucial role in this change.

Between 2009 and 2022, beavers created up to 25 ponds. The number of ponds varied each year, in the range from 11 (in 2022) to 25 (in 2017). The number of migration corridors ranged from 4 (in 2009) to 20 (in 2020). Until 2016, there was an increase in the number of corridors, but then it fluctuated. The total surface area of the ponds is more interesting, as it indicates the water storage capacity. The total sum of the surface of the ponds ranged between 1067.6 m^2^ in 2019 and 2215.78 m^2^ in 2016. The statistically significant increase in surface was between 2015 and 2016 (Wilcoxon test, *p* < 0.05).

A characteristic feature of the landscape without and with beavers, visible when comparing vegetation maps, is the change in the patchiness of the vegetation and the dominant habitat group (Figure 2 and Figure 3). Although the total number of communities decreased slightly, the number of patches (PR) increased greatly (Table 6). At the same time, the area of the dominant community increased, resulting in a high LPI and a lower evenness index. The patchiness of the landscape increased after the release of the beavers when using the vegetation maps as the base (Table 6).

## 4. Discussion

The analysis of vegetation changes during the long-term presence of beavers is quite challenging, primarily due to the lack of sufficient data from the period before the beavers were released. Consequently, research on this topic is conducted rather rarely [19,63,64,65]. However, in recent years, researchers have begun to study beaver meadows and areas that have experienced beaver-caused floods [32,33,34,35]. Our understanding of the reintroduction process, along with evidence of the long-term presence of beavers and some historical data, enabled us to conduct research in the mountain stream in the protected area of the Syhłowaciec stream valley in the Bieszczadzki NP (Eastern Carpathians, Poland).

However, we faced certain limitations. The first limitation was the absence of historical phytosociological relevés from before the reintroduction of beavers, which prevented us from determining whether the species composition of plant communities had become enriched or impoverished. The second limitation involved difficulty in accessing historical data, such as vegetation maps and orthophotomaps from the same periods, which hindered our ability to make direct comparisons, although some observations were still possible. General knowledge indicates that this valley is occupied by one family of beavers. Furthermore, we did not investigate aquatic communities; therefore, our list does not include macrophyte communities that may have formed in beaver ponds. However, individual reports suggest that the aquatic environment could serve as a habitat for new species emerging in this area [43,45].

Analysis of vegetation maps in the Syhłowaciec stream valley over three time periods revealed significant changes in vegetation, particularly in the first period after the introduction of beavers. These changes in habitat conditions led to the disappearance of some plant communities and the formation of new ones that had not existed previously in the valley. The presence of beaver ponds facilitated the development of the horsetail community Equisetetum fluviatilis. Furthermore, the Phalarido-Petasitetum hybridi community also emerged as a result of beaver activity. Both of these plant associations experienced periodic flooding, creating favourable conditions for their development and survival. Certain plant species and communities thrive in environments with high soil moisture or bodies of water, such as aquatic or semi-aquatic macrophytes [24,64]. Hydrological changes, such as water retention and alterations in groundwater levels [26,31,32], greatly influence plant species composition and vegetation. Consequently, changes in the development, disappearance, or coverage of plant communities can be documented. The continuous presence of beavers, along with the pressure they exert on tree and shrub species, has resulted in the decline or disappearance of communities with grey alder. This decline may be linked to beavers gathering materials for dam-building and foraging during winter [65,66,67]. The search for food and building materials can be linked to the increased number of migration corridors over time, as seen in our study. Furthermore, the continued presence of beavers in the stream valley has hindered the restoration of forest communities and the establishment of other thickets. In addition to the disappearance and development of new communities, transformations were observed among plant communities. For example, the fen Valeriano-Caricetum flavae transformed into a community with *Menyanthes trifoliata* and Scirpetum sylvatici. Such transformations due to the presence and activity of beavers have been documented in other regions [31,68]. It can be concluded that beaver activities do indeed transform plant communities; however, the specific changes depend on the area and the existing vegetation, making it essential to evaluate each case individually.

Changes in the Syhłowaciec stream valley and the transformations of plant communities may also be linked to previously observed alterations in soil moisture and the prevalence of moisture-loving species [26,27,40]. Soil moisture in the valley may have increased due to the presence of beaver migration corridors, which have varied over the years. These corridors could also facilitate plant dispersal. Research has shown that beavers modify the structure of riparian vegetation and species composition [15,16,69].

Using two methods, vegetation studies and satellite imagery, allowed us to examine the impact of beavers on stream landscapes from a different perspective. By combining the results of both approaches, we observed changes in the beaver meadows. Other studies suggest that these meadows develop in former beaver ponds where sediment accumulates over time [70,71]. Our results indicate that in the upper part of the valley, between 1996 and 2010, part of the eutrophic fen Valeriano-Caricetum flavae was flooded. After the water receded (post-2010), the area became overgrown with moist and wet communities from the Molinio-Arrhenatheretea class. Over time, the landscape heterogeneity changed. The dynamic mosaic of moist and wet communities can be observed with increased patchiness. Recently, it has been well documented that disturbance related to beaver activity results in landscape patchiness and supports a variety of species and ecological processes [34,35].

Four years of field observations provided additional insight. Following the dam failure and receding water, the first community to develop in the former beaver pond was the horsetail community Equisetetum fluviatilis. Since succession is a gradual process that depends on the species present in these areas [6,70,72], we speculate that the next community to appear may be the sedge community Caricetum rostratae, as *Carex rostrata* plays a significant role in the development of wetland areas.

Satellite images serve as an effective tool for analysing landscape changes due to the availability of orthophotomaps from multiple sources. Our results reveal that beavers significantly alter the landscape, and access to these orthophotomaps allows for the observation of changes over many years. Although few studies have examined increases in stream bed length, some authors have observed differences in the shape and length of watercourses [22,73,74]. It was observed in a small stream of an upper tributary of the Tamar River in southeast England, with three years of beaver activity, that the length of the stream increased from 180 m to 210 m [23]. In addition, some authors have documented changes in the number of beaver ponds over the years [16,72,73,74,75]. In Hudson Bay, Canada, the authors noticed a fluctuation in the total area of ponding between 1985 and 2021, from a decrease by 53% to 80% by 1995, which then recovered to initial levels by 2015. This fluctuation was linked with the size of the beaver population [76]. Integrating these changes with colonisation data offers a more comprehensive perspective [15,16,17].

The changes in vegetation and landscape observed in the Syhłowaciec stream valley were directly linked to the presence of beavers and their activity. However, it cannot be ruled out that ongoing climate change also influences succession processes, although the rate of plant response to these changes is certainly slower [77]. For the park area, an increase in average temperature and precipitation over the last 40 years has been documented [49,78], and in the longer term, changes in vegetation [79].

## 5. Conclusions

Beavers significantly influence their environment, particularly in areas close to their habitats. This effect is particularly notable in mountain streams, where their presence may not be immediately obvious. Their activities, such as building dams, lead to notable landscape changes, including the creation of ponds, the number of which can vary from year to year. An indirect impact of beavers is alterations in the length of the streambed; in the mountain stream area studied, the length increased by 9.5%, or 81 m, due to the formation of meanders. The ponds created by beavers can exceed 2200 m^2^, contributing importantly to local humidity levels. As water accumulates behind the dams, soil moisture increases, resulting in changes in plant species composition and vegetation around the watercourse. Additionally, the presence of beaver migration corridors, which fluctuate in number from year to year, can enhance soil moisture in the valley. The increased number of corridors can also be linked with the search for food, storage, and building materials. These corridors may also facilitate the plant dispersal. The increased soil moisture promotes the development of wet and moist plant communities that would not exist without beavers. Consequently, the significance of these moist and wet plant communities is growing; in our study area, 89% of the terrain was dominated by such vegetation in 2021. Our findings offer a detailed perspective on the sequence of vegetation and landscape changes following the introduction of beavers. This information could be valuable in understanding environmental changes caused by beavers in both protected areas and those affected by human activity.

## Figures and Tables

**Table 1:** Coverage [ha] of plant communities in the Syhłowaciec stream valley, Bieszczadzki NP, Eastern Carpathians. 1996—before beaver release (source of map: BdNP archive); 2010—14 years with beaver activity [51], 2021—25 years with beaver activity. EUNIS habitat codes after Chytrý et al. (2020) [62].

Plant Community	EUNIS Habitat Code	Habitat Group	1996	2010	2021
PHRAGMITETEA					
Equisetetum fluviatilis	Q51	wet	-	0.08	0.07
Caricetum paniculatae	Q53	wet	0.01	0.01	0.1
Caricetum rostratae	Q53	wet	0.05	0.16	0.4
Sparganio-Glycerietum fluitantis	Q52	wet	-	0.01	0.01
		*total*	*0.06*	*0.25*	*0.58*
MOLINIO-ARRHENATHERETEA					
Filipendulo-Geranietum	R55	wet	1.11	0.63	2.71
Scirpetum sylvatici	R35	wet	0.08	0.23	0.3
		*total*	*1.19*	*0.86*	*3.01*
Cirsietum rivularis	R35	moist	0.36	0.09	0.19
Epilobio-Juncetum effusi	R35	moist	0.49	0.39	-
Community with *Mentha longifolia*	R35	moist	0.21	-	0.04
		*total*	*1.06*	*0.48*	*0.23*
Community with *Deschampsia caespitosa*	R35	fresh	0.13	0.06	0.05
Campanulo serratae-Agrostietum typicum	R22	fresh	-	0.28	-
Campanulo serratae-Agrostietum capillaris alopecuretosum pratensis	R22	fresh	0.91	1.01	0.65
		*total*	*1.04*	*1.35*	*0.7*
SCHEUCHZERIO-CARICETEA NIGRAE					
Caricetum diandrae	Q41	wet	0.19	-	-
Carici canescentis-Agrostietum caninae	Q24	wet	-	0.03	-
Valeriano-Caricetum flavae	Q41	wet	1.56	1.57	-
Community with *Menyanthes trifoliata*	Q41	wet	-	0.85	0.3
		*total*	*1.75*	*2.45*	*0.3*
ARTEMISIETEA VULGARIS					
Phalarido-Petasitetum hybridi	R55	wet	-	-	0.08
Anthriscetum sylvestris	R55	moist	-	-	0.09
Community with *Urtica dioica*	R55	fresh	0.31	-	0.01
BETULO-ADENOSTYLETEA					
Poo-Deschampsietum caespitosae	R56	fresh	0.04	-	-
QUERCO-FAGETEA					
Caltho laetae-Alnetum	T12	wet	0.81	-	-
Others					
Community with *Agropyron repens*	R22	fresh	0.17	-	-
Community with *Calamagrostis canescens*	R22	fresh	0.01	-	-
Community with *Vaccinium myrtillus*	T41	fresh	-	0.03	-
		*total*	*0.18*	*0.03*	*0*
Community with *Carex brizoides*	R22	moist	0.09	0.3	0.76
Community with *Alnus incana* on agricultural land	T12	moist	0.25	-	-
Thickets and tree groups	T12	moist	-	1.06	1.03
		*total*	*0.34*	*1.36*	*1.79*

**Table 2:** Characteristic of vegetation, the Syhłowaciec stream valley, Bieszczadzki NP, Eastern Carpathians. 1996—before beaver release (source of map: BdNP archive); 2010—14 years with beaver activity [52], 2021—25 years with beaver activity.

Characteristic of Vegetation	1996	2010	2021
area of vegetation studied [ha]	6.78	6.79	6.79
total area of fresh plant communities [ha]	1.57 *	1.38	0.71 *
total area of moist plant communities [ha]	1.4 *	1.84	2.11 *
total area of wet plant communities [ha]	3.81	3.57	3.97
area of fresh plant communities [%]	23.15	20.32	10.46
area of moist plant communities [%]	20.65	27.09	31.07
area of wet plant communities [%]	56.19	52.58	58.47
number of plant communities	18	17	16
—continuing		10	10
—extinct (against 1996)		8	8
—new (against 1996)		7	6
changes between 2010 and 2021			
—continuing between 2010 and 2021			12
—extinct between 2010 and 2021			5
—new between 2010 and 2021			4

*—*p* < 0.05 between years (Wilcoxon test).

**Table 3:** Discriminant analysis of environmental variables measured with EIV (n = 89, 12 groups; three plots representing their own communities were omitted in the analysis).

	Lambda	Part	F	*p*	Toler.	1-Toler.
Tr	0.2293	0.2339	23.523	0.0000	0.847	0.152
L	0.0541	0.1941	22.354	0.0000	0.740	0.259
T	0.0142	0.2240	19.129	0.0000	0.765	0.234
W	0.0043	0.3077	15.368	0.0000	0.726	0.273
R	0.0021	0.5027	6.564	0.0000	0.822	0.177

**Table 4:** Changes in length [m], number of beaver ponds, and migration corridors of the Syhłowaciec stream, BdNP, Eastern Carpathians.

Year	Length [m]	Beaver Ponds	Migration Corridors
1969	837	-	-
1994	837	-	-
2009	838	14	4
2015	870	22	9
2016	900	20	20
2017	897	25	12
2019	895	16	8
2020	912	14	20
2021 **	915 **	13	11
2022	918	11	9

** the orthophotomap of that period did not cover the entire length of the stream.

**Table 5:** Surface [m^2^] of beaver ponds in the Syhłowaciec stream valley in 2009–2021 based on orthophotomaps on a scale of 1:2500. No data—orthophotomap incomplete, -—no pond.

Pond/Year	2009	2015	2016	2017	2019	2021
1	14.4	46.94	116.57	135.17	46.15	no data
2	-	25.89	22.25	15.58	7.56	no data
3	-	-	5.9	15.76	-	no data
4	374.24	300.64	306.67	281.34	210.67	-
5	188.52	115.12	-	-	-	-
6	86.8	333.4	-	-	-	-
7	355.77	309.81	353.72	322.2	184.6	55
8	31.75	482.65	581.49	232.6	257.6	490.6
9	162.26	105.75	167.13	-	-	79.9
10	-	17.44	42.48	67.31	74.5	116.8
11	-	31.26	57.35	67.47	18.66	62.44
12	32.66	-	-	-	-	-
13	11.13	34.92	51.37	66.91	18.7	71,14
14	18	6.99	34.34	25.58	46.74	92.9
15	18.34	6.65	15.83	6.7	-	16.8
16	9.17	-	42.6	9.52	-	-
17	14	8.01		6.88	-	-
18	-	-	4.49	4.94	6.77	75.5
19	300.05	86.64	372.88	55.56	123.4	208.67
20	-	17.7	46.82	43.23	25.56	64.13
21	-	6.27	36.49	23.06	46.69	25.7
SUM	1617.09	1936.08	2215.78	1379.81	1067.6	1295.45

**Table 6:** Landscape features based on vegetation maps and satellite images between 1996 and 2021 in the Syhłowaciec stream valley, Bieszczadzki NP, Eastern Carpathians. 1996—before beaver release (source of map: BdNP archive); 2010—14 years with beaver activity [51], 2021—25 years with beaver activity.

Landscape Feature	1996	2010	2021
stream length (m)	837	838	915
number of beaver ponds	-	14	13
number of migration corridors	-	4	11
area of ponds surface [m^2^]	-	1617.09	1295.45
number of habitat groups	3	3	3
number of communities	18	17	16
area of fresh communities [%]	23.15	20.32	10.46
area of moist and wet communities [%]	76.84	79.67	89.54
area with *Alnus incana* [%]	15.63	0	0
Patch Richness	29	42	57
Largest Patch Index [%]	16.8	15.5	39.9
Shannon–Wiener Index	2.492	2.391	1.928
Shannon’s Evenness	0.846	0.827	0.712

## Data Availability

The original contributions presented in this study are included in the article. Further inquiries can be directed to the corresponding author.
